# MIRAGE Syndrome Caused by a *De Novo* c.3406G>C (p. Glu1136Gln) Mutation in the *SAMD9* Gene Presenting With Neonatal Adrenal Insufficiency and Recurrent Intussusception: A Case Report

**DOI:** 10.3389/fendo.2021.742495

**Published:** 2021-09-29

**Authors:** Xinyi Chin, Aravind Venkatesh Sreedharan, Ene Choo Tan, Heming Wei, Jyn Ling Kuan, Christopher Wen Wei Ho, Joyce Ching Mei Lam, Teck Wah Ting, Rashida Farhad Vasanwala

**Affiliations:** ^1^ Department of Paediatric Medicine, Endocrinology Service, KK Women’s and Children’s Hospital, Singapore, Singapore; ^2^ KK Research Centre, KK Women’s and Children’s Hospital, Singapore, Singapore; ^3^ SingHealth Duke-NUS Paediatric Academic Clinical Programme, Singapore, Singapore; ^4^ SingHealth Duke-NUS Institute of Precision Medicine (PRISM), Singapore, Singapore; ^5^ Department of Paediatric Medicine, Gastroenterology Hepatology & Nutrition Service, KK Women’s and Children’s Hospital, Singapore, Singapore; ^6^ Department of Paediatric Subspecialties, Haematology/Oncology Service, KK Women’s and Children’s Hospital, Singapore, Singapore; ^7^ Department of Paediatric Medicine, Genetics Service, KK Women’s and Children’s Hospital, Singapore, Singapore

**Keywords:** MIRAGE, primary adrenal insufficiency, adrenal hypoplasia, intussesception, SAMD9/SAMD9 mutations

## Abstract

**Introduction:**

Primary adrenal insufficiency (PAI) presenting in the neonatal period can be life threatening and requires early recognition, diagnosis, and management. PAI due to adrenal hypoplasia (syndromic/non-syndromic) is a rare disorder. MIRAGE is a recently described syndrome with PAI and multisystem involvement.

**Case Presentation:**

A preterm female neonate presenting with PAI and persistent severe thrombocytopenia was diagnosed to have MIRAGE syndrome due to a *de novo* pathogenic variant c.3406G>C (p. Glu1136Gln) in the *SAMD9* gene. In the first year of life, she had recurrent respiratory and gastrointestinal infection causing failure to thrive. At 17 months, she suffered recurrent intussusception requiring treatment with parenteral nutrition and high-dose steroids. Subsequently, she established oral feeds with hydrolysed formula and demonstrated good weight gain.

**Conclusion:**

In neonates presenting with PAI and associated multisystem involvement, a thoughtful approach and genetic testing is valuable in discerning an etiological diagnosis. This case of MIRAGE adds to the spectrum of reported cases and is the first to report on recurrent intussusception and its management with high-dose steroids.

## Introduction

Adrenal hypoplasia due to syndromic or non-syndromic aetiology is a rare cause of primary adrenal insufficiency (PAI). MIRAGE is a recently described multisystemic disorder with adrenal hypoplasia which has been added to the spectrum of syndromic causes of PAI presenting in early life. MIRAGE syndrome is caused by heterozygous activating mutations in the sterile alpha motif domain-containing 9 (*SAMD9*) gene located on chromosome 7q which encodes a protein involved in growth factor signal transduction. The mutation leads to antiproliferative effects resulting in multisystem growth restriction. The six prominent phenotypes include Myelodysplasia, Infections, Restriction of growth, Adrenal hypoplasia, Genital phenotypes, and Enteropathy (OMIM #617053) (1). It is a very rare condition with only around 40 cases reported (2) with variable phenotypic expressions. Most of the SAMD9 mutations occur *de novo* except in two reported families where it was transmitted from asymptomatic mothers (3, 4). We present here a case of MIRAGE syndrome in a girl with *de novo* mutation c.3406G>C and describe the unique features of this case as well as the presentation of recurrent intussusception which has not been previously reported.

## Case Description

### Neonatal

Our patient was born at 30 + 1 weeks to healthy, non-consanguineous parents. The family history was unremarkable, and her two older siblings were well. She was born normal for gestational age with weight of 1,145 g (-0.59 SDS), length of 38 cm (-0.25 SDS), and OFC of 26.5 cm (-0.4 SDS). She was intubated at birth in view of respiratory distress, required pressor infusions, and escalation to broad-spectrum antibiotics (meropenem was added to first-line penicillin G and gentamicin) for presumed sepsis. Pancytopenia was present at birth with haemoglobin of 8 g/dl, leucocytes of 4.03 × 10^9^/l, and platelets of 14 × 10^9^/l, necessitating transfusion. There was a brief period of clinical stability before her haemodynamics deteriorated again at day 6 of life due to a symptomatic patent ductus arteriosus. Attempts were made to pharmacologically close the PDA, which was eventually successful after two rounds of medications (indomethacin followed by ibuprofen). Concurrently in the second week of life, she developed hyponatremia (128 mmol/l) and hyperkalemia (6.9 mmol/l), and subsequently hyperpigmentation was noted around the third week of life which prompted a referral to endocrinology. On examination, the child had generalized hyperpigmentation out of proportion for her Chinese ethnicity, no dysmorphic features, normal female external genitalia without virilisation, and a normal systemic examination except for a systolic murmur. A short synacthen test revealed baseline elevated ACTH >1337 ng/l, peak stimulated cortisol of 200 nmol/l (normal >500 nmol/l), and a normal 17-hydroxyprogesterone level of 96 ng/dl (normal <630 ng/dl) (**Table 1**). Ultrasound abdomen showed hypoplastic adrenal glands. These findings were consistent with PAI. She was started on hydrocortisone (25 mg/m^2^/day) and sodium supplementation (4 mmol/kg/day), with improvement of the electrolytes and hyperpigmentation. In view of persistent thrombocytopenia in conjunction with PAI, genetic testing to rule out syndromic and non-syndromic causes was done at 6 weeks. Written informed consent was obtained from parents. Genomic DNA was extracted from cultured dermal fibroblasts derived from the patient’s skin and tested on the TruSight™ One Sequencing Panel. A *de novo* heterozygous missense variant of SAMD9 [NM_017654.3: c.3406G>C (p. Glu1136Gln)] was detected.

**Table 1:** Laboratory data.

Variables	Results	Reference range	Variables at 3 weeks	Results	Reference range
Hb					
Day 1	8.0	14.0–22.5 g/dl	Sodium	128	133–146 mmol/l
3 months	10.9	11.5–15.5 g/dl	Potassium	6.9	3.7–5.9 mmol/l
28 months	12.1	11.0–16.0 g/dl	ACTH	>1337	10.0–60.0 ng/l
WBC					
Day 1	4.03	9.0–30.010^9^/l	Cortisol	186 (0 min)	Peak > 500 nmol/l
3 months	5.74	5.0–15.010^9^/l		200 (30 min)	
28 months	4.5	4.0–10.010^9^/l		198 (60 min)	
Plt					
Day 1	14	140–44010^9^/l	17-OHP	94 (0 min)	<630 ng/dl
3 months	141	140–44010^9^/l		96 (30 min)	
28 months	80	140–44010^9^/l		73 (60 min)	
			Aldosterone	424.1	97.3–834 pmol/l
			Active Renin	>350	3.6–20.1 pg/ml
			DHEA-S	0.65	28.9->40.7 μmol/l
			Testosterone	0.6	0.04–2.15 nmol/l

Hb, haemoglobin; WBC, white blood cells; Plt, platelets; ACTH, adrenocorticotropic hormone; 17-OHP, 17-hydroxyprogesterone; DHEA-S, dehydroepiandrosterone sulphate.

### Post-Neonatal Up to 1 Year

**Figure 1:**
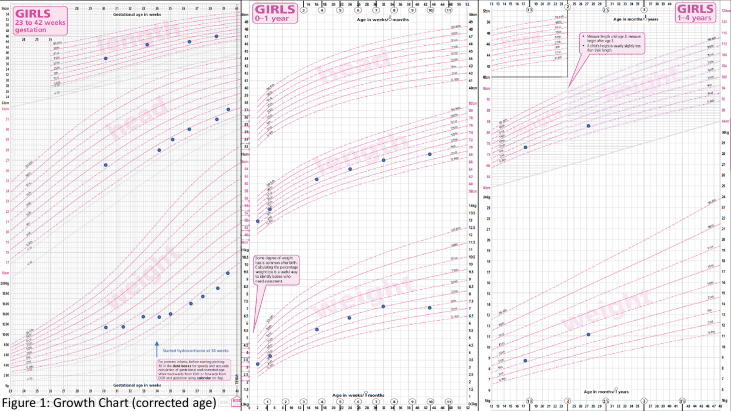
Growth Chart (corrected age).

Postnatal growth was miserly, and she dropped from the 25th percentile to below the second percentile for both weight and length centile in the first 10 weeks. Neither bottling of feeds nor direct latching was successfully established. A video fluoroscopic swallow study (VFSS) done at 3 months of life demonstrated moderate oropharyngeal dysphagia; as such, the child remained on nasogastric tube feeding. She was given a combination of high-caloric milk and expressed breast milk, which was well-tolerated, without any diarrhoea. Following this, she had improvement in growth with both weight and height centile inching back to the 25th percentile. The need for weekly platelet transfusion ceased at 3 months, and she was discharged home stable. At this juncture, anaemia had resolved with a persistence of moderate thrombocytopenia. At age of 8 months, she was treated for *E. coli* UTI. This was soon followed by a period of profuse non-bloody diarrhoea secondary to Norovirus infection. This was treated by complete gut rest and intravenous hydration followed by gradual introduction of an extensively hydrolysed, lactose-free, hypoallergenic formula. She was again admitted after 2 months for separate episodes of bronchiolitis (nasopharyngeal aspirates positive for respiratory syncytial virus and parainfluenza), requiring bronchodilator therapy and oxygen supplementation. During each admission, profuse diarrhoea up to 10 episodes/day was observed. Her parents reported that the bowel output reverted to a daily baseline of three to five episodes of pasty stools in between each illness. In view of the recurrent respiratory and gastrointestinal infections in the first year, extensive immunology workup was done, and she was found to have only mild B cell lymphopenia with no significant immunocompromise at baseline. She recorded normal immunoglobulin response to vaccines. Due to recurrent infections, she again dropped her centile from 25th to 9th and subsequently has maintained along the 9th percentile for both weight and height (**Figure 1**). A repeat VFSS done at 1 year of age showed great improvement of the oropharyngeal dysphagia, her acceptance of solid food remained poor, and she was given pureed feeds *via* syringe by her mother. As such, she remained on a predominantly milk-based diet by mouth. Due to repeated bouts of intercurrent infection and feeding challenges, she had intermittent faltering growth with catch-down followed by catch-up during the first year.

### 1–2 Years

**Figure 2:**
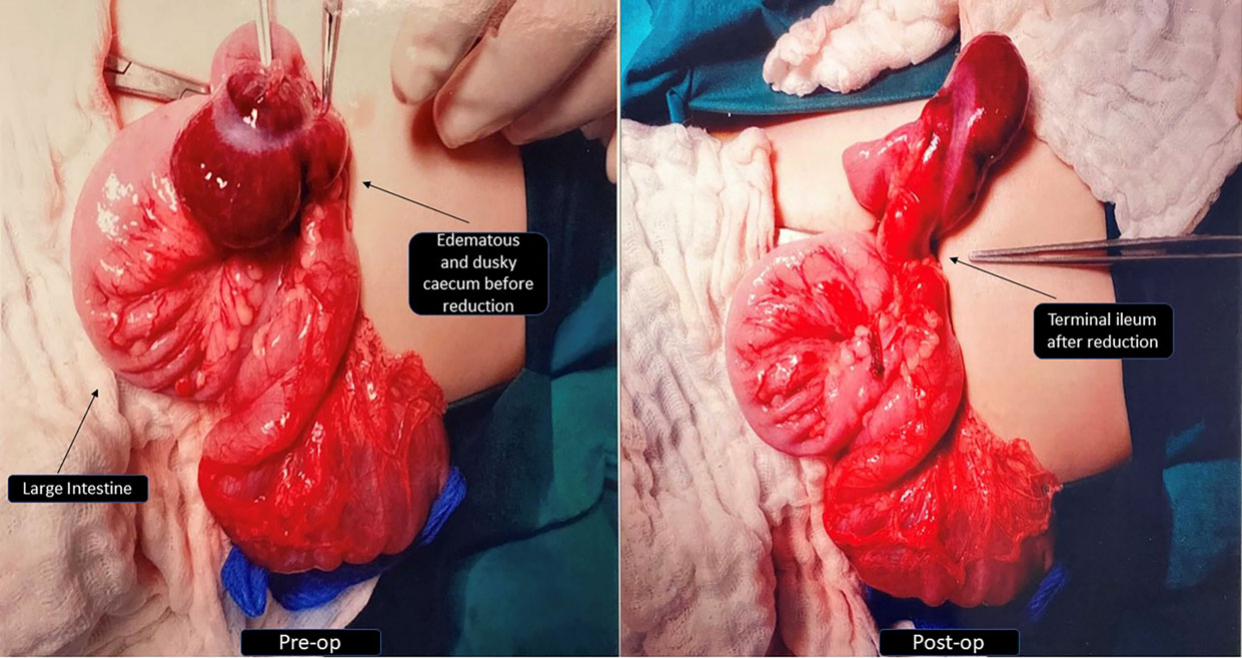
Intra-operative photos before and after reduction of ileo-colic intussusception.

**Figure 3:**
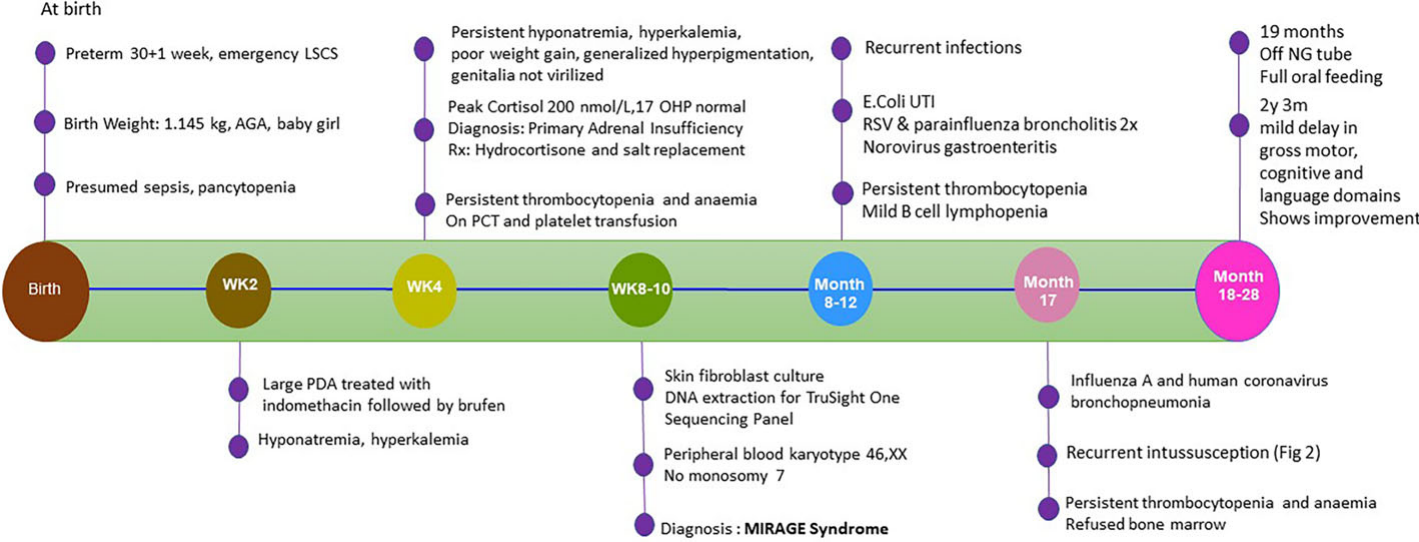
Timeline of events from birth until 28 months.

Up until 17 months, her growth was slow but steady and both weight and height were maintained at the ninth percentile as there were no further intercurrent infection, oral feeding had improved, and bowel output was normal. Then came the stormiest course at 17 months of age, where she first developed bronchopneumonia (nasopharyngeal aspirate positive for Influenza A and human coronavirus). Soon after, she experienced three episodes of intussusception within a week, each heralded by currant-jelly stools and haemodynamic instability. The first episode was an ileo-colic intussusception which required manual reduction *via* open laparotomy after a failed laparoscopic attempt. Intraoperative findings revealed a tight long segment of intussusception up to the splenic flexure, and no obvious lead point was identified. Intestinal biopsy was not performed while attempting to reduce the intussusceptum surgically since the bowel was noted to be edematous and at risk of perforation (**Figure 2**). A gastroenterology referral was sought, and she was placed on strict gut rest and total parenteral nutrition. The subsequent two episodes were colo-colic intussusception amenable to air-enema reduction. At this juncture, she was experiencing profuse bloody diarrhoea of more than 10 episodes a day causing anaemia (haemoglobin nadir of 6.8 g/dl, platelet nadir of 18 × 10^9^/l), which was thought to be due to a combination of reactive myelosuppression and ongoing blood loss. It is interesting to note that the location of the intussusception along the large bowel differed each time and there was no lead point. Serial ultrasounds of the abdomen revealed diffuse colon thickening (maximal thickness of 9.4 mm) and areas suggestive of pneumatosis. The likely cause of intussusception could be the thickened bowel from intercurrent infection possibly contributed by underlying enteropathy even though she did not have chronic diarrhoea. In view of pancolitis and recurrence of the second and third episodes of intussusception despite complete gut rest, decision was made for a trial of IV methylprednisolone (1.6 mg/kg/q24h). Thereafter, there was no further recurrence of intussusception and the blood in stools started to resolve. The dose of methylprednisolone was gradually weaned over the course of 3 weeks. Stress dose hydrocortisone was titrated in tandem with the clinical status as well as during the period of methylprednisolone administration. Parents declined diagnostic endoscopy and biopsy. The inpatient stay was further prolonged when she developed line-related Bacillus sp. bacteremia 3 weeks after the last episode of intussusception. She was discharged on nasogastric tube feeding with extensively hydrolysed milk formula. **Figure 3** provides a timeline of events.

### Post 2-Year–Follow-Up

At present, she is 28 months old; there has been no further hospitalisation. With regard to feeding, she was gradually reintroduced to solids and normal milk formula, which is now well-tolerated by mouth. Nasogastric tube was removed at the age of 19 months. Stool output is reported to be Bristol type 4 with a daily frequency of three to five times. On serial full blood count monitoring, haemoglobin remained stable between 10.8 and 14.1 g/dl, white cells 4.4–7.4 × 10^9^/l (no neutropenia), and platelets 31–46 × 10^9^/l. Although chronic thrombocytopenia appears to be the main cell line involved, there has been no clinical bleeding manifestation. Serial examinations of the peripheral blood film have not shown any dysplastic changes. A bone marrow examination has not been conducted due to parental refusal. The last peripheral blood karyotype done at 1 year of age did not show presence of monosomy 7. Her dose of hydrocortisone is maintained at 15 mg/m^2^/day. Growth has been channelling, and her weight at 27 months was 11.2 kg (25th percentile) and height of 82.6 cm (2nd-9th percentile) (**Figure 1**). A formal developmental assessment (Bayley III) at 2 years and 3 months showed mild delay in the gross motor, cognitive, and language domains. She has since been enrolled into an early intervention program. Her overall intellectual development is normal and on follow-up noted improvement in her motor and language skills as well.

## Discussion

### MIRAGE, a Newly Described Syndrome Associated With Adrenal Hypoplasia

Previously known syndromes associated with adrenal hypoplasia include triple AAA syndrome, IMAGe (CDKN1C), IMAGe-I (POLE1), SERKAL syndrome (WNT4), and syndrome with MCM4 mutations, with MIRAGE added in 2016 (1, 5). The term MIRAGE syndrome was first coined in 2016 after Narumi et al. identified 11 patients with adrenal hypoplasia sharing similar extra-adrenal features who were found to have mutations in causative gene *SAMD9*. Subsequent reports served to expand on the phenotypic features of this syndrome (2). PAI is a highly consistent feature, seen in 94% of patients from the two larger cohorts. Except for the few exceptions in cases without adrenal insufficiency (3, 4), we believe that PAI is almost pathognomonic in MIRAGE syndrome given that *SAMD9* expression is found to be highest in the foetal adrenal gland. Postmortem biopsies revealed hypoplastic glands with dysgenetic adrenocortical cells (1, 6). Although at present no formal diagnostic criteria have been established for MIRAGE syndrome, we believe that our patient suffers from this condition based on what has been proposed by Tanase-Nakao et al., 2020 (2).

### Unique Features of Our Case

We report a case MIRAGE syndrome with a *de novo* heterozygous missense variant of *SAMD9* [NM_017654.3: c.3406G>C (p. Glu1136Gln)] detected in exon 2. To our knowledge, the *SAMD9* variant c.3406G>C (p.Glu1136Gln) found in our patient has not been previously described in repertoire of pathogenic variants accounting for MIRAGE syndrome. This variant is not present in population databases. It is also absent in her parents. This variant was reported in the literature in a family with multiple affected individuals (7), although the phenotype was mainly myelodysplastic syndrome and incomplete penetrance was noted. The variant was predicted to be deleterious and polymorphism by SIFT and Mutation Taster, respectively. As such, we classified this variant as a likely pathogenic variant based on published guidelines of American College of Medical Genetics and Genomics (ACMG) and the Association of Molecular Pathology (AMP). We propose that the *SAMD9 variant c.3406G>C* detected in our case has a different phenotypic expression as compared to that reported by Schwartz et al. (7). He reported a germline *SAMD9* mutation in siblings with monosomy 7 and myelodysplastic syndrome. The three siblings reported were diagnosed with refractory cytopenias of childhood based on cytogenetics and bone marrow findings. All three siblings had transient thrombocytopenia in the neonatal period requiring platelet transfusion. Two out of three siblings were male, and one was born with severe hypospadias and bifid scrotum requiring corrective surgeries. The two oldest siblings have been treated with bone marrow transplants while the youngest remains asymptomatic with normal peripheral blood counts. The loss of chromosome 7 was confirmed in the myeloid cells of all three siblings. The genetic analyses pointed to a heterozygous missense mutation (c.3406G>C) in *SAMD9* resulting in p.E1136Q mutation as the causal lesion for MDS and monosomy 7 in this family. Sanger sequencing of genomic DNA from peripheral blood of parents confirmed presence of mutation in the asymptomatic mother, confirming this as an inherited variant. The siblings do not have any other associated features of MIRAGE syndrome. Although our patient has persistent thrombocytopenia, so far, she has not shown presence of monosomy 7 or developed any features of dysplasia in the peripheral blood film. Also, unlike the three siblings she has other features of MIRAGE. The entire spectrum from asymptomatic mother to isolated MDS in two out of three affected offspring to MIRAGE syndrome in our patient underscores the current appreciation of the lack of clear genotype–phenotype correlations.

We believe that our patient exhibits a milder MIRAGE phenotype as evidenced by 1) absence of intrauterine growth restriction, 2) initial postnatal growth restriction followed by catch-up growth unlike the severe persistent growth restriction reported in MIRAGE, 3) adrenal insufficiency which is adequately managed without need of fludrocortisone, 4) history of infections which were not particularly invasive, 5) absence of overt enteropathy, 6) normal intellectual development, and 7) relatively good quality of life.

Several observed or proposed mechanisms have been put forth to account for such a vast variability seen in other cases of MIRAGE syndrome. In a case series of eight patients, Buonocore et al. (6) first reported that a more severe phenotype was observed in patients whose *SAMD9* variant involved an arginine residue compared to those possessing non-arginine residues (6). Rentes et al. (8) proposed that variants in the TPR repeat domain of *SAMD9* are less damaging when they drew the parallel between the 9-year-old patient and those with familial MDS described by Schwartz et al. (7, 8). This is of particular interest to us, as our patient share the same p.E1136Q variant.

To escape the growth-restrictive effect of the mutation, there can be a compensatory mechanism with progressive loss of the mutated allele (monosomy 7 or 7q), termed as adaptation-by-aneuploidy and/or appearance of a somatic “second hit” negating the gain of function (6). This mechanism rescues the severe phenotype and explains the wide spectrum of disease presentation, although the acquisition of monosomy 7 also increases the risk of myelodysplasia. How and when the adaptation occurs is not known. The spatiotemporal events (adrenal gland, then haematopoietic system, gastrointestinal tract, and so on) could be explained by a stem cell niche repopulation in which an initial somatic reversion mutation had occurred. The earlier the reversion occurs during embryogenesis, the more likely chance that revertant descendant stem cells spread as large clusters, resulting in milder phenotypes (9, 10).

### Enteropathy

The enteropathy in MIRAGE syndrome most commonly presents as chronic non-infectious diarrhoea (2, 4), often needing parenteral nutrition in the face of failure to thrive and protein-losing enteropathy. Many of the patients faced feeding difficulties requiring nasogastric tube or percutaneous endoscopic gastrotomy. Neither endoscopy nor biopsy findings shed further light; as such, the mechanism resulting in enteropathy remains unknown. Recurrent aspiration pneumonia has been attributed to oesophageal problems, including oesophageal hypoperistalsis and achalasia (11). It is to our best knowledge that this is the first case of recurrent multifocal intussusception in a child with MIRAGE syndrome. In the majority of childhood intussusception, there is no clear disease trigger or pathologic lead point. Idiopathic intussusception is most common in children between 3 months and 5 years of age with the ileo-colic junction being the most common site of occurrence (12). Viral triggers are suggested to play a role in idiopathic intussusception due to lymphoid hyperplasia, especially in the lymphoid-rich terminal ileum. The reported overall intussusception recurrence rate is 7.5% to 12.7% (13), and factors associated with recurrent intussusception are age more than 1 year, duration of symptoms (≤12 h), absence of vomiting, abdominal mass located in right abdomen, and presence of a pathological lead point (14). When recurrent intussusception occurs in association with lymphoid hyperplasia and no other lead point can be identified, treatment with glucocorticoids has been suggested to prevent recurrence (15).

There was a preceding history of viral infection in our patient, but her atypical presentation of recurrent multifocal intussusception without lead point raises the possibility of enteropathy related to her genetic syndrome. The likely cause of intussusception could be the thickened bowel wall from intercurrent infection and possibly contributed by chronic enteropathy even though she did not have chronic diarrhoea. We speculate that occurrence of aggravated bowel output with each infective episode might suggest an element of chronic subclinical mucosal inflammation. Also, *SAMD9*, which functions in endosome fusion and regulates cell growth in mutated state, will affect cell repair and regeneration. The gut being a high cell turnover organ, this function will be hugely affected, leading to chronic tissue damage without repair and lead to atrophy. Therefore, it is important to monitor for evolving enteropathy even in an asymptomatic patient without diarrhoea. This inference can be drawn from our case, and she will need close monitoring of gut health.

## Conclusion

This is the first case of recurrent multifocal intussusception reported in a child with MIRAGE syndrome. A trial of high-dose steroid was attempted, and it averted further recurrence. Although this is not a novel mutation, this child does not bear any phenotypic resemblance with the cluster of isolated familial MDS. Also, she has less severe growth restriction, normal intellectual development, and good quality of life. Adrenal insufficiency is a primary feature of this syndrome, and any neonate with adrenal insufficiency and hematologic/immunologic/genital abnormalities should be screened for *SAMD9* gene mutation. We hope to contribute these unique features to the growing repertoire of phenotypic spectrum of the disease to better understand this rare condition.

## Data Availability Statement

The original contributions presented in the study are included in the article/supplementary material. Further inquiries can be directed to the corresponding author.

## Ethics Statement

Written informed consent was obtained from the individual(s) for the publication of any potentially identifiable images or data included in this article.

## Author Contributions

XC and RF were involved in the conception of the work and writing and are the main authors of the manuscript. RF also critically reviewed and revised the manuscript. AS contributed in the writing. TT, ET, HW, and JK were involved in the genetic testing and also contributed in writing the genetic methodology and results. JL provided the haematology and CH gastroenterology input in case management. All authors approved the final document and are accountable for all aspects of the work. All authors contributed to the article and approved the submitted version.

## Funding

Genetic analysis with the TruSight™ One Sequencing Panel is supported by a Centre Grant (NMRC Project No. NMRC/CG/M003/2017) from the National Medical Research Council, Ministry of Health, Republic of Singapore.

## Conflict of Interest

The authors declare that the research was conducted in the absence of any commercial or financial relationships that could be construed as a potential conflict of interest.

## Publisher’s Note

All claims expressed in this article are solely those of the authors and do not necessarily represent those of their affiliated organizations, or those of the publisher, the editors and the reviewers. Any product that may be evaluated in this article, or claim that may be made by its manufacturer, is not guaranteed or endorsed by the publisher.
